# CIVIC ENGAGEMENT IN LATER LIFE: USING THE GERONTOLOGICAL IMAGINATION TO DEVELOP RESEARCH ON MIGRANTS

**DOI:** 10.1093/geroni/igae098.1676

**Published:** 2024-12-31

**Authors:** Sandra Torres, Pernilla Agard

**Affiliations:** Uppsala University, Uppsala, Uppsala Lan, Sweden; Uppsala university, Uppsala, Uppsala Lan, Sweden

## Abstract

Social gerontologists that have brought attention to the knowledge gaps that exist in research on late-life-civic engagement have already proposed that this research has not yet exploited the theorizing potential embedded in migratory life-courses. In a similar fashion research on migrants’ civic engagement has not yet envisioned that aging and old age offer interesting vantage points from which that research can be expanded. This presentation taps into both of these states of affairs by bringing attention to what characterizes the scholarly imagination on migrants’ civic engagement, the taken-for-granted assumptions about this engagement that this literature makes, and the areas of research that have yet to receive attention. Through the systematic coding of the peer-reviewed literature that focuses on migrants’ civic engagement this presentation aims to offer insights into, among others, the kinds of research questions that this literature is preoccupied with, the angles of investigation and methodological approaches that it relies on as well as the types of civic engagement, and the taken-for-granted assumptions about them, that the literature never problematizes. In doing so, we will argue that the gerontological imagination (especially if combined with migrancy-astuteness) could expand the ways in which migrants’ civic engagement have been conceived and measured thus far.

